# Supplementary material for: Measuring the impact of nonpharmaceutical interventions on the SARS-CoV-2 pandemic at a city level: An agent-based computational modelling study of the City of Natal
Source: PLOS Glob Public Health. 2022 Oct 14;2(10):e0000540. doi: 10.1371/journal.pgph.0000540 (PMC10021960; doi:10.1371/journal.pgph.0000540)
Supplement: Attachment — Submitted filename: Response to reviewers comments.pdf [file pgph.0000540.s002.pdf]

PGPH-D-22-00736R2

Response to reviewers' comments for the manuscript: "Measuring the impact of nonpharmaceutical interventions on the SARS-CoV-2 pandemic at a city level: An agent-based computational modelling study of the City of Natal"

Editor Comments #1:

[E1MC1] How did you deal with differences between government policies and adherence to them, from all the actors involved, considering that they also had different behaviors during the pandemic that affected this adherence. Real data were affected by these behaviors, and in some cases they could be different according to their places of residence, age and socioeconomic status. Apparently, simulation did not consider this.

[ANSWER] We agree with the Editor that population adherence is critical to implementing government policies. Unfortunately, we found no data that allowed us to assess the population adherence to the guidelines differentially according to the agent's features, such as age, place of residence or work, and socioeconomic status. We decided not to analyze the many possible hypothetical scenarios of differential adherence as the additional complexity would probably be more confusing than informative and would likely express some level of prejudice. Still, there are significant indications of widespread commitment to the policies, as indicated by the overall reduction of mobility reported by local telecom companies and few reports of commerce opening in the local news outlets. In the specific scenario of the decrees that affected the opening of business and religious places, we included the not optimal adherence, as reported in the media, by allowing a value different than zero. Thus, the search process for the baseline scenario indirectly accounted for the overall level of adherence specific to that layer.

We acknowledge the limitation in our analysis in a new paragraph added to the discussion (page 27, line 616).

[E1MC2] You mentioned that the "incubated state follows a log-normal distribution". It is not understandable why did you include estimated means and standard deviations of death values, assuming that they were normal. In fact, in some of the figures, means and standard deviations are far away from real data. How do you interpret these differences in Figure 5A, 5B, 6B, 8A.

[ANSWER] We thank the reviewers for the remarks. We have now modified all displays to show the median, and 5%, 25%, 75% and 95% percentiles. These displays

show the statistics of multiple simulations with the same parameters but different random seeds.

The factual data, as shown, is subject to noise in the reporting process. For instance, Sundays tend to have fewer reports, while Monday and Tuesdays tend to display a higher number of reports. We did not model this noise source, which explains why the real data have higher variability than the simulations. Still, as we focus on the cumulative curve of events for the search of parameters, this noise has little effect on the setting of the model.

We have updated the discussion to indicate these limitations of the simulations (page 27, line 629).

Reviewer #1:

The manuscript describes use of an agent-based model to assess intervention strategies used during the COVID-19 pandemic. I have two major concerns:

[R1MC1] The authors do not describe how they optimized the model to fit the data used for validation. They need to explain which parameters were adjusted to fit the data and what algorithm was used. The baseline curve they ended up with underestimates the cumulative deaths for a period of time. It's also not clear if the government decrees were incorporated into the fit by changing parameters at the time the decrees went into effect. Since the baseline parameters help determine the relative importance of different interactions in spreading the virus, having incorrect baseline values will lead to incorrect predictions of the effect of different interventions.

[ANSWER] Thank you for your comments. We apologize for not including in the first version of the manuscript a clear description of the method to obtain the baseline parameters.

We conducted an exploratory search in the model parametric space. The parameters included in the baseline scenario - subject to the search - are the initial P contamination and the specific reduction in the P contamination specific to each decree. The time of the decrees was fixed and determined by the actual date they were published. Therefore, we incorporated the government decrees into the fit.

The evaluation of each baseline candidate included the R-square difference between the actual and the simulated cumulative death curve and the absolute difference in the estimated deaths. At first, a brute approximation from randomly selected parameters ended once a candidate reached an R-square level above 0.95 and the absolute

difference in the cumulative deaths was below 10%. Next, we conducted a local search by computing all neighbouring scenarios with parametric steps of +/- 0.8, 0.5, 0.2, and 0.1. For this step, we averaged the result of 30 simulations for each parametric set. We then selected the parametric set with the R-square value above 0.99 with the lowest difference in the cumulative death count. To validate the baseline scenario, we ran the simulation another 500 times and obtained an average R-square value of 0.9927 and an underestimation of just one cumulative death.

We have now updated the text in the Methods and Results to reflect our methodology to estimate the baseline parametric set (page 17 line 352; page 19 line 417) and added a more detailed description to the Supplementary Material.

The obtained baseline scenario has the limitation of underestimating the cumulative death curve for a certain period, which indicates that we did not find a global maximum in the search or that the level of abstraction of the model cannot account for a small aspect of the actual phenomena. Still, we are confident that these limitations do not hinder our conclusions as the overall quality of the fit is good. We have updated the discussion to indicate these limitations of the model and list possible aspects not considered in our work (page 27, line 627).

[R1MC2] A global sensitivity analysis might be more useful here to assess which classes or sub-classes contribute most to the number of fatalities.

[ANSWER] As advised by the Reviewer, we ran a global sensitivity analysis and added the description to the text in the Results section (page 18 line 375).

In our analysis, we run the model without restrictions with all layers set with the same Pcontamination value (Pcontamination = 1.7). We then run new simulations after reducing the P contamination of each network individually (Pcontamination = 1.5). By comparing the total reduction of cumulative deaths recorded in each of the manipulations, we can glimpse each layer's global impact.

[R1MC3] The figures are of low resolution and need to be improved. It's also not clear what the inset graph on the final graphs of figures 6, 7, and 8 is supposed to represent.

[ANSWER] Thank you for highlighting this. We submitted the figures with a low resolution to comply with the file size limit established by the submission system. For this review, we submitted the figures in a vector format to ensure the reviewers could access the figures in full resolution.

The inset graph in figures 6 and 7 represents the impact in life saved of an eventual delay (in weeks) of the application of the decree described in the first panel of each figure (6A - Work - and 7A - Churches).

We applied the same methodology to the inserted graph in figure 5 (Schools layers simulation scenarios). We updated the labels in all Figures to enhance clarity.

[R1MC4] Line 8: The 18 million deaths mentioned here is rather high compared to the 6 million or so officially recorded COVID deaths to date. The authors should make clear that the 18 million figure is based on excess mortality estimates and that not all these deaths are directly attributable to COVID.

[ANSWER] Thank you for your remark. We have updated the Introduction with the official WHO death toll for 2021 and also mentioned the excess death values as an alternative estimation. The text now reads:

> By the end of 2021, the pandemic had claimed the lives of about 5.4 million people worldwide, according to the WHO, or up to 18 million causalities when considering excess death estimates.

[R1MC5] The description of adding external infections is not entirely clear. Are these new agents added to the model or are the appropriate number of new infections randomly assigned to the existing population?

[ANSWER] Thank you for pointing this out. The appropriate number of new infections (total of 3957 calculated in equation 2) are randomly assigned to the existing population of Natal city (total of 873383). We updated the explanation of the addition of external infections in the Methods section (page 16 line 331).

[R1MC6] - Line 501: One of the two dates (June 19th or June 16th) is incorrect, since the curve cannot reach the peak on the 19th and "remain there" to three days before it reaches the peak.

[ANSWER] The reviewer is correct. The second date should be July 16th. The text was updated with the correct date.

[R1MC7] - While the English is generally clear, there are places where incorrect terminology or wording is used. The manuscript should be checked by a native English speaker.

[ANSWER] Thank you for the suggestion. We have copyedited the new version of the manuscript with a native English speaker.

Reviewer #2:

Overall, this is an interesting application study. The evaluation of the effect of governmental measures in order to estimate their effects and to help to prepare for future events is really important.

We thank the reviewer for the comments.

[R2MC1] While the modeling strategy seems similar to other models in the literature (which makes its novelty weaker), I see the results presented interesting. It would make a reacher contribution to put into perspective the reason why one would like to extend the SIR model and what is the effect of this extra complexity in the quality of the modeling and also to put it into the context of other similar studies. Right now the paper presents no discussion or justification for this.

[ANSWER] We would like to thank the reviewer for the comments and suggestions. The agent-based approach to epidemiology modelling is not entirely novel and was explored before and during the pandemic. Our main contribution is to express how one can adapt the model to the realm of data available within one city. Agent-based modelling is reasonably fit for quickly including many aspects that could be relevant

(or not) for a valuable portrait of the pandemic dissemination within the city. The cost of having such versatility with agent-based models is the loss of the generalization and computational efficiency of traditional compartmental models. Adding new aspects to a compartment-based SIR model is possible, but not without a higher toll on the design cost and the mean-field approach's reliability. Creating new compartments for representing different aspects of the data will reduce the number of events associated with each compartment and lower its approximation accuracy. Notably, the relative simplicity of adding new aspects and features to an agent-based model has its costs as an explosion of possibilities lowers the interpretability of the simulations. Therefore, we focus our modelling on the aspects we could sustain with data that should be commonly available in municipalities, which, in our view, is the main novelty of our work.

We have improved the introduction to better position our modelling approach within the current state-of-the-art and justify our modelling selections (page 5, line 84). We have also added an extended discussion about the model selection, limitations and advantages of our decisions and comparison with other SIR models in the discussion (page 26, line 607).

[R2MC2] In particular, on page 11 the paper introduces equation 1 that has this variable Pcontamination (which the equation uses a small p for pcontamination, while the text uses capital P).

[ANSWER] Thank you for pointing this out. All variables "P contamination" were changed to capital P.

[R2MC3] On page 17, subsection Decrees, the phrase "We did not set the Pcontamination value to zero because some establishments continued to work" seems strange to me. Since, if on is allowed to set Pcontamination to a low value, this would automatically say that the decrees were effective, which I believe it is something that one wants to validate and fit from the data. So I believe this has to be made clear in the paper.

[ANSWER] Thank you for highlighting this. We did not zero the Pcontamination for the Work Layer Decree because there were reports of not a total commitment to the Decree [1]. The value reported, above zero and below a normal functioning of commerce generally, was subject to the search algorithm. We have updated the

description of why Pcontamination value was set differently than other values for the Religion or Education layers, for example (page 15 line 298).

[1] https://g1.globo.com/rn/rio-grande-do-norte/noticia/2020/03/28/feiras-livres-voltam-a-acontecer-em-natal-apos-uma-semana-de-suspensao-por-causa-do-coronavirus.ghtml

[R2MC4] Other criticism concerning the paper is related to its organization: in many points the paper is verbose and repeats itself in multiple occasions, there is in many places text. The figures are now well organized, being convoluted with too much information and hard to interpret at times.

[ANSWER] Thank you. We have revised the text for conciseness with special attention to the results section.

[R2MC5] I would suggest that these figures should be reworked especially figure 2, from which it is hard to extract information from and figures 6 and 7.

[ANSWER] Thank you for the suggestion. We have reorganized Figure 2 by reducing the number of panels (we now only show selected networks and not all networks) and by adding a label to better explain the network plots. The objective of the figure is to display to the reader how diverse were different networks, which is now clearly stated in the label.

We have also reorganized figures 5, 6 and 7 by repositioning the labels to express more clearly that groups of panels reflect different simulation scenarios. We have also modified some labels to simplify the understanding of the reader.

[R2MC6] Finally, there are many numbers concerning the data from the city and its different areas that I believe could be condensed in a table, instead of being scattered though the paper

[ANSWER] Thank you for pointing this out. We have condensed all demographic data into a signle tabel (Table 1).
