# Supplementary material for: Measuring the impact of nonpharmaceutical interventions on the SARS-CoV-2 pandemic at a city level: An agent-based computational modelling study of the City of Natal
Source: PLOS Glob Public Health. 2022 Oct 14;2(10):e0000540. doi: 10.1371/journal.pgph.0000540 (PMC10021960; doi:10.1371/journal.pgph.0000540)
Supplement: Attachment — Submitted filename: Response to the Editor.docx [file pgph.0000540.s001.docx]

**Response to the Editor**

Journal Requirements:

1. Please amend your detailed Financial Disclosure statement. This is published with the article. It must therefore be completed in full sentences and contain the exact wording you wish to be published.

State what role the funders took in the study. If the funders had no role in your study, please state: “The funders had no role in study design, data collection and analysis, decision to publish, or preparation of the manuscript.”

- The funders had no role in study design, data collection and analysis, decision to publish, or preparation of the manuscript added to the Financial Disclosure statement

2. Please send a completed 'Competing Interests' statement, including any COIs declared by your co-authors. If you have no competing interests to declare, please state “The authors have declared that no competing interests exist”. Otherwise please declare all competing interests beginning with the statement "I have read the journal's policy and the authors of this manuscript have the following competing interests:

- “The authors have declared that no competing interests exist” added to 'Competing Interests' statement

3. Please ensure that you provide a single, cohesive .tex source file for your LaTeX revision. You may upload this file as the item type 'LaTeX Source File.' As stated in the PLOS template, your references should be included in your .tex file (not submitted separately as .bib or .bbl). Please also ensure that you are making any formatting changes to both your .tex file and the PDF of your manuscript. If you have any questions, please contact [Latex@plos.org](mailto:Latex@plos.org). You can find our LaTeX guidelines here: 
<https://journals.plos.org/globalpublichealth/s/latex> "

- LATEX file added to the submission with bibliography info added to single .tex file

4. Please provide separate figure files in .tif or .eps format and remove any figures embedded in your manuscript file. Please also ensure that all files are under our size limit of 10MB. If you are using LaTeX, you do not need to remove embedded figures.

For more information about how to convert your figure files please see our guidelines: <https://journals.plos.org/globalpublichealth/s/figures>

- All figures submitted separately and converted to TIFF using PLoS System

5. Please provide an Author Summary. This should appear in your manuscript between the Abstract (if applicable) and the Introduction, and should be 150–200 words long. The aim should be to make your findings accessible to a wide audience that includes both scientists and non-scientists. Sample summaries can be found on our website under Submission Guidelines: <https://journals.plos.org/globalpublichealth/s/submission-guidelines#loc-parts-of-a-submission>
Alternative link: <http://journals.plos.org/ploscompbiol/s/submission-guidelines#loc-author-summary>

- Author Summary added to the manuscript text

6. We notice that your supplementary tables are included in the manuscript file. Please remove them and upload them with the file type 'Supporting Information'. Please ensure that each Supporting Information file has a legend listed in the manuscript after the references list.

- Supplementary Tables put in a separate file

Additional Editor Comments (if provided):

Dear authors

We appreciate the submission of your paper to our journal. Even when the way you submitted the manuscript let us to know how it is going to be its layout versión, it is not the appropriate one, that is suggested in the "Instructions for Authors". It is important to follow this Instructions, given that it makes easier the the reviewers' work. At the end of your submission you could add, your "layout version".

- Manuscript adjusted according to the Layout and spacing instruction on the Instruction to Authors page.
